# An Adaptive Low-Cost GNSS/MEMS-IMU Tightly-Coupled Integration System with Aiding Measurement in a GNSS Signal-Challenged Environment

**DOI:** 10.3390/s150923953

**Published:** 2015-09-18

**Authors:** Qifan Zhou, Hai Zhang, You Li, Zheng Li

**Affiliations:** 1 Beihang University, No. 37 Xueyuan Road, Haidian District, Beijing 100191, China; E-Mails: zhanghai@buaa.edu.cn (H.Z.), bayexx@126.com (Z.L.); 2 University of Calgary, 2500 University Drive N.W. Calgary, AL T2N1N4, Canada; E-Mail: liyou331@gmail.com

**Keywords:** tightly-coupled integration, adaptive Kalman filter, GPS, MEMS-IMU

## Abstract

The main aim of this paper is to develop a low-cost GNSS/MEMS-IMU tightly-coupled integration system with aiding information that can provide reliable position solutions when the GNSS signal is challenged such that less than four satellites are visible in a harsh environment. To achieve this goal, we introduce an adaptive tightly-coupled integration system with height and heading aiding (ATCA). This approach adopts a novel redundant measurement noise estimation method for an adaptive Kalman filter application and also augments external measurements in the filter to aid the position solutions, as well as uses different filters to deal with various situations. On the one hand, the adaptive Kalman filter makes use of the redundant measurement system’s difference sequence to estimate and tune noise variance instead of employing a traditional innovation sequence to avoid coupling with the state vector error. On the other hand, this method uses the external height and heading angle as auxiliary references and establishes a model for the measurement equation in the filter. In the meantime, it also changes the effective filter online based on the number of tracked satellites. These measures have increasingly enhanced the position constraints and the system observability, improved the computational efficiency and have led to a good result. Both simulated and practical experiments have been carried out, and the results demonstrate that the proposed method is effective at limiting the system errors when there are less than four visible satellites, providing a satisfactory navigation solution.

## 1. Introduction

Inertial navigation systems (INS) and global navigation satellite systems (GNSS) have been widely used to provide accurate and reliable navigation information (*i.e.*, attitude, velocity and position). GNSS has long-term stability in ideal conditions, but has certain limitations in urban areas (e.g., a city downtown), inside tunnels and under heavy tree canopies. INS is completely self-contained and autonomous, but suffers from accuracy degradation over time. The integration of GNSS and INS can maximize their respective advantages, minimize their individual drawbacks and provide a more satisfactory navigation solution. Especially in recent years, Micro-Electro-Mechanical System (MEMS) sensors have met the specifications and requirements needed for applications in various fields because of their low power consumption, small size, light weight and low cost. Accordingly, low-cost GNSS/INS integration systems have become an increasingly attractive option. In most commercial GNSS/INS products, the GNSS-derived positions and velocities are integrated with MEMS sensors through a Kalman filter (KF) for the navigation solution [1,2]. In the meantime, the IMU is also used to provide the navigation information during GNSS signal outages and can be used for fast GNSS signal reacquisition [3,4]. A precondition for utilizing such loosely-coupled integration methods is that at least four satellites are visible. However, this premise is not always satisfied, especially in urban areas. If less than four satellites are visible, the filter cannot be updated, because the GNSS position is unavailable.

Several studies have focused on providing continuous and accurate position results with less than four satellites [5,6]. The common approach is to use dead-reckoning with inertial sensors and other sensors to bridge GNSS outages. To mitigate the drifts of dead-reckoning, several methods have been presented. One direct method is to make use of external sensors or equipment, such as magnetometers or an odometer, to provide heading or velocity updates [7,8] when GNSS is invalid. Moreover, cameras and laser scanners can be used to extract the features of scanned objects for position and heading determination in urban scenarios when the satellite number is below four [9,10]. This kind of method is effective, but relies on additional sensors, which are not always affordable. Another popular and attractive method to solve the navigation problem with less than four satellites is the non-holonomic constraints (NHC) [11,12]. This method takes advantage of the knowledge of the vehicle’s dynamics and the physical conditions that the vehicle experiences. This knowledge is utilized as measurements in the vehicle state estimation process [13]. However, this method can only be applied when constrained conditions exist during the dynamic process.

Additional literature has contributed to enhancing the attitude estimation without the need for extra hardware. Examples of these methods include: using nonlinear estimation algorithms [14] or using neural networks; enhancing the quality of sensor data through calibration [15], stochastic modeling [16] and de-noising [17]; and introducing *a priori* information, such as control inputs, vehicle maneuver models and kinematic constraints [18]. Additionally, some other methods, such as post-processing with a Rauch-Tung-Striebel (RTS) smoother, provide more reliable results [19] and are always used in applications for which it is not critical to obtain the position solution in real time, like mobile mapping.

However, these methods have their limitations and, thus, only work under specific situations. On the one hand, methods based on additional sensors (laser scanner, camera) are effective, but are not always affordable for civilian navigation applications and cannot properly provide real-time solutions. On the other hand, approaches that utilize *a priori* information can improve the navigation performance for some applications under specific scenarios; however, these methods cannot completely solve the divergence of the navigation errors. The most important point is that these listed methods do not consider or evaluate the quality of measurement in the process. Because the situation in which a reduced number of visible satellites occurs most often when the signal is heavily blocked, the remaining visible satellite measurements will properly have a lower performance that affects the filter solution if we do not adopt approaches that adjust the filter parameters.

In order to solve the listed problem and to achieve the main objective of this paper, which limits the system errors when the GNSS signal is challenged, such that less than four satellites are visible in a harsh environments, we put forward an adaptive tightly-coupled integration system with height and heading aiding (ATCA), which adopts an adaptive noise covariance tuning strategy and combines the external aiding measurement in both a directly and pseudo measurement aiding manner. The main contributions of our research include:

1. We present a novel adaptive method to tune the Kalman filter measurement noise covariance matrix® in real time online and mitigate the effect of GNSS measurement errors caused by the changing of visible satellites. The proposed method has the advantage that the tuning process is dependent on only measurements and is totally decoupled from estimated state vectors.
2. This research suggests using information from external sensors to enhance the navigation performance, and the whole system works under a filter switching strategy. This means that when at least four satellites are visible, the system works in standard tightly-coupled mode without employing the external measurements in order to improve the computational efficiency; when three satellites are visible and the barometer data are available, the system switches to the height aiding filter; and when two satellites are available and the magnetometer and height information are also available, the system changes to the height/heading aiding integration filter.
3. We utilize both the barometer and magnetometer measurements, not only in a directly aiding manner, but also a pseudo-measurement and velocity measurement manner. Specifically, we present the method of using the height measurement by approximately modeling the Earth as a static pseudo satellite; also, the magnetometer measurements are used to aid the velocity measurement, which implicitly assumes that the receiver moves in the direction of its heading, which actually is an implicit NHC approach. The benefit is that the measurements are more deeply coupled with the indirect related states in the Kalman filter. For example, the height measurements can even be potentially correlated with the horizontal position errors; also, the magnetometer measurements may enhance not only the INS heading, but also the horizontal velocities.

This paper is organized as follows: Section 2 describes the standard GNSS/INS tightly-coupled method; Section 3 introduces the proposed method, including the overall system design; Section 4 illustrates the theorem and the proof of the method for adaptively tuning the measurement noises and the utilization of this approach in a GNSS/INS tightly-coupled system. Section 5 provides the height and heading measurements aiding the filter; Section 6 gives the design and implementation of the hardware platform of the tightly-coupled integration system. Section 7 provides both simulation and real test results, and Section 8 finally draws the conclusions.

## 2. Standard Tightly-Coupled GNSS/INS Integration

**Figure 1:**
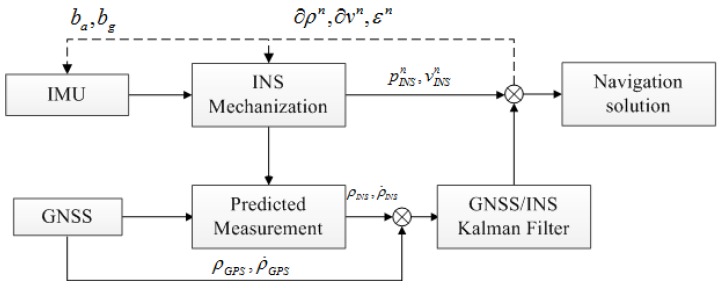
Tightly-coupled integration scheme.

Before introducing the proposed ATCA method, we describe the standard tightly-coupled GNSS and INS integration system here first, and its block diagram is shown in Figure 1. As shown in Figure 1, the GNSS/INS Kalman filter processes the difference of GNSS output and INS derived pseudo-ranges and pseudo-rates as measurement directly and corrects the INS mechanization in a closed loop with the estimation results. The Kalman filter state (system) and measurement equations are described separately in this section.

### 2.1. Tightly-Coupled System State Model

The tightly-coupled integration system uses the INS error propagation model as its dynamic model. The state vector is composed of the INS navigation errors (*i.e.*, attitude errors, velocity errors and position errors), IMU sensor errors (e.g., gyroscope and accelerometer biases) and the distance error caused by clock errors (e.g., clock bias and clock drift) of the GNSS receiver. The state vector is given as follows:

(1) $$\begin{array}{l} X=[X_{INS}\;X_{IMU}\;X_{GPS}]\\ X_{INS}=[\phi_{E},\phi_{N},\phi_{U},\delta v_{E},\delta v_{N},\delta v_{U},\delta L,\delta \lambda ,\delta h]\\ X_{IMU}=[\varepsilon_{x},\varepsilon_{y},\varepsilon_{z},\nabla_{x},\nabla_{y},\nabla_{z}]\\ X_{GPS}=\left[cdt\;cd\dot{t}\;\right] \end{array}$$

where $\left[\phi_{E},\phi_{N},\phi_{U}\right]$, $\left[\delta v_{E},\delta v_{N},\delta v_{U}\right]$ and [δL,δλ,δh] denote the errors of attitude, velocity and position, respectively; $\left[\varepsilon_{x},\varepsilon_{y},\varepsilon_{z}\right]$ and $\left[\nabla_{x},\nabla_{y},\nabla_{z}\right]$ represent gyroscope and accelerometer biases; cdt and $cd\dot{t}\;$ denote the distance error caused by the receiver clock bias and clock drift, respectively. The subscripts *E*, *N* and *U* denote the east, north and up components in the local navigation frame (*l*-frame), and the subscripts *x*, *y* and *z* denote the right, front and up components in the body frame (*b*-frame).

Then, according to the chosen state vector, the system dynamic model can be written as follows:

(2) $$\begin{array}{l} \dot{X}=F(t)X(t)+\Gamma (t)W(t)\\ \;=\left[\begin{array}{ccc} {\left(F_{I}\right)}_{9\times 9} & {\left(F_{S}\right)}_{9\times 6} & 0_{9\times 2}\\ 0_{6\times 9} & {\left(F_{M}\right)}_{6\times 6} & 0_{9\times 2}\\ 0_{2\times 9} & 0_{2\times 6} & {\left(F_{G}\right)}_{2\times 2} \end{array}\right]\left[\begin{array}{c} X_{ins}(t)\\ X_{imu}(t)\\ X_{gps}(t) \end{array}\right]+\left[\begin{array}{ccc} \Gamma_{I}(t) & 0_{9\times 6} & 0_{9\times 2}\\ 0_{6\times 6} & I_{6\times 6} & 0_{6\times 2}\\ 0_{2\times 6} & 0_{2\times 6} & \;I_{2\times 2} \end{array}\right]\left[\begin{array}{c} W_{1}(t)\\ W_{2}(t)\\ W_{3}(t) \end{array}\right] \end{array}$$

where 0 and I separately denote the zero and unity matrix. Several literature works [20,21] have described the INS error-based dynamic model; the model is not illustrated here specifically, but the detailed description of the system model is listed in Appendix for reference.

### 2.2. Tightly-Coupled System Measurement Model

In the GNSS/INS tightly-coupled integration system, the differences between GNSS-measured and INS-derived pseudo-ranges and pseudo-rates are taken as observations for the filter:

(3) $$Z(t)=\left[\begin{array}{l} Z_{\rho }(t)\\ Z_{\dot{\rho }}(t) \end{array}\right]=\left[\begin{array}{l} \rho_{GPS}-\rho_{INS}\\ {\dot{\rho }}_{GPS}-{\dot{\rho }}_{INS} \end{array}\right]=\left[\begin{array}{l} H_{\rho }\\ H_{\dot{\rho }} \end{array}\right]X(t)+V(t)$$

where $\rho_{GPS}$ and ${\dot{\rho }}_{GPS}$ denote the GNSS pseudo-range and pseudo-rate and $\rho_{INS}$ and ${\dot{\rho }}_{INS}$ denote the pseudo-range and pseudo-rate predicted from the satellite and INS motion information. $H_{\rho }$ and $H_{\dot{\rho }}$ denote the measurement matrices. *V* is the measurement noises and is white, zero-mean, uncorrelated and has the covariance matrix $E[V_{k}V_{j}^{T}]=R_{k}\delta_{k-j}$. Because the measurement equation has also been introduced in several literature works [22,23] and is not illustrated here specifically, the detailed description of the measurement model is listed in Appendix for reference.

Although the standard tightly-coupled GNSS/INS integration system is designed specifically for navigation scenarios with less than four satellites, the filter performance degrades dramatically because of the lack of measurement information [24]. Two reasons mainly contribute to the performance degradation. The first one is that the observability matrix is not a full column rank matrix, and the system becomes unobservable when the visible satellite number is less than four [25]. In this situation, the rank of the null space is greater than one, so the existing measurement information cannot estimate the exact INS system error, and the final navigation solution’s error has an accumulating trend. In other word, the rest of the satellite information is not strong enough to provide the position constraints. The second reason is the negative effect of the measurement error. Typically, the less than four satellites situation most often happens when the GPS signal is heavily blocked, such as driving downtown. The block that causes the invisibility of the majority of the satellites will in the meantime lead to the remaining visible satellites suffering from large measurement error. Then, in the measurement update of the Kalman filter, the inaccurate satellite measurement information unfortunately further influences the system state estimation. Especially when a low-cost IMU is used, the positioning error may increase rapidly and even lead to the divergence of the filter.

## 3. Overview of the Proposed System

Based on the drawback analyses of the standard tightly-coupled system previously described, aiming to solve the current existing problems, the main idea to solve the problem is, first, adding external auxiliary measurements, such as height and heading, to increase the system observability and to provide stronger position and velocity constraints; second, adopting the adaptive measurement noise estimation to have the evaluation of the measurement quality and optimally blend the data from the GNSS and INS.

**Figure 2:**
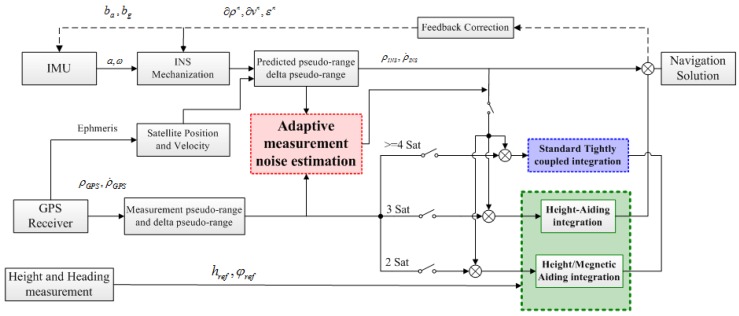
Adaptive tightly-coupled aiding (ATCA) system scheme.

Hence, the proposed ATCA method mainly focuses on the following points: (1) adopt an adaptive Kalman filter to adjust the measurement noise covariance matrix (*R*) in real time based on the situation, instead of using a constant matrix, to reflect the noise characteristics accurately; (2) employ external auxiliary measurements effectively; and, finally; (3) redesign the filter for different situations and switch them in the navigation process. The system scheme is shown in Figure 2.

In the ATCA system, first, an adaptive measurement noise estimation is introduced, which is shown in the red block, and it makes use of the two different sources of pseudo-range and pseudo-rate to estimate measurement noise variance and then adjusts it online. Compared to traditional methods, which are based on the innovation sequence [26,27], this adaptive method takes only GNSS measurements and INS predictions into consideration. Therefore, this method is totally decoupled from estimated state vectors.

Second, if at least four satellites are visible, the ATCA system works in the traditional tightly-integrated mode and combines the GNSS-measured pseudo-range and pseudo-rate with INS to construct the measurement vectors, as described in Section 2 before, and this part is shown in the blue block in Figure 2. The filter utilizes the residuals to obtain the INS error estimates and corrects the navigation components in a closed loop.

Finally, when fewer satellites are visible, the ATCA system works in aiding mode, and this part is shown in the green block in Figure 2. In detail, when only three satellites are available, the system introduces an external height measurement and switches to the height-aiding mode. A new algorithm is presented in this mode, and it treats the Earth as a static pseudo satellite and adds an ellipsoid equation-based position constraint into the measurement equations. Meanwhile, when only two satellites are available, the system changes to height/heading integration aiding mode; the magnetometer information is introduced and used in a velocity measurement aiding manner to correct not only the heading, but also the horizontal velocities. The main reason to adopt the switch filter strategy is to decrease the system computational burden for the real-time navigation application. We build the prototype of our tightly-coupled integration system aiming to provide a real-time navigation solution in practice, so the system computational efficiency is also a significant point that needs to be considered in the design process. With the filter switch strategy, we only involve the aiding information when less than four satellites are available, and the measurement matrix will have a lower dimension in the more than four satellites available periods, so the filter computation will be decreased in such a situation. As referred to in [28], if we only consider the multiplication computation here, the multiplication number for the one-step recursion of the Kalman filter is $2n^{3}+3n^{2}m+n^{2}+2nm^{2}+2nm$. Hence, if the height and heading measurements are all included in the measurement equations (*n* = 17, *m* = 12) all of the time, the multiplication number is 25,823 for the one-step recursion, while the number is only 19,499 during the more than four visible satellites period (*n* = 17, *m* = 8) with the filter switching strategy. Therefore, we can save 24.5% multiplication operations for the one-step recursion of Kalman filter. Moreover, we do not have to utilize a blunder detector to evaluate the measurement of the barometer and magnetometer in each epoch to save the computational resources. Furthermore, we do not need to collect the height and heading information from the external equipment through the I2C (Inter-Intergrated Circuit) or UART (Universal Asynchronous Receiver/Transmitter) interface in every sample epoch to save the system hardware resources, and the savings will become obvious when the system requires a higher measurement sampling frequency.

## 4. Adaptive Kalman Filter

Real-world navigation scenarios are complex and unpredictable. For example, it is a common case that some satellites are blocked or becoming invisible. When one satellite is losing its sight, the satellite pseudo-range and pseudo-rate errors will increase significantly and even become unacceptable before the satellite becomes invisible. Moreover, the visible satellites may suffer from large pseudo-range and pseudo-rate errors. In other word, the real measurement noise is strongly dependent on the navigation scenarios. However, in many applications, it is difficult to predict the navigation environment, and hence, the Kalman filter itself is preferred to be smart enough to tune the measurement noise covariance adaptively according to the navigation environment and measurement quality.

In order to solve this problem, the adaptive Kalman filter is the most commonly-used method and can be found in several literature works [29,30]. However, this method is always an innovation sequence-based adaptive estimation (IAE) approach and will involve the sate vector *X* during the calculation of the measurement noise covariance. Therefore, if the state is not well estimated, a negative effect properly occurs for the filter performance. Here, in order to avoid such risks, a novel adaptive method is introduced based on the redundant measurement system noise estimation theorem. Both the theorem and proof of the proposed method are provided in this section.

### 4.1. Theorem: Noise Estimation Based on Redundant Measurement Systems

Assume that $Z_{1}(k)$ and $Z_{2}(k)$ are measurements of the value Z from different systems at time *k*. Here, the measurements from System 1 and System 2 can be expressed as follows:

(4) $$\left\{ \begin{array}{l} Z_{1}(k)=Z(k)-\left[f_{1}(k)+V_{1}(k)\right]\\ Z_{2}(k)=Z(k)-\left[f_{2}(k)+V_{2}(k)\right] \end{array}\right.$$

where $V_{1}(k)$ and $V_{2}(k)$ are independent and zero mean white noises, $f_{1}(k)$ and $f_{2}(k)$ are trend items of the measurement errors. If the following conditions are satisfied:

(5) $$\left\{ \begin{array}{l} diag\left[\begin{array}{ccc} \cdots & {\left(f_{1}^{i}(k)-f_{1}^{i}(k-1)\right)}^{2} & \cdots \end{array}\right]\ll E[V_{1}(k)V_{1}{\left(k\right)}^{T}]\\ diag\left[\begin{array}{ccc} \cdots & {\left(f_{2}^{i}(k)-f_{2}^{i}(k-1)\right)}^{2} & \cdots \end{array}\right]\ll E[V_{1}(k)V_{1}{\left(k\right)}^{T}]\\ E[V_{2}(k)V_{2}{\left(k\right)}^{T}]\ll E[V_{1}(k)V_{1}{\left(k\right)}^{T}] \end{array}\right.$$

The measurement noise variance of System 1 can be estimated as:

(6) $$R_{1}=E\left[V_{1}(k)V_{1}{\left(k\right)}^{T}\right]\approx E\left\{\left[\Delta Z_{1}(k)-\Delta Z_{2}(k)\right]{\left[\Delta Z_{1}(k)-\Delta Z_{2}(k)\right]}^{T}\right\}/2$$

where:

(7) $$\left\{ \begin{array}{l} \Delta Z_{1}(k)=Z_{1}(k)-Z_{1}(k-1)\\ \Delta Z_{2}(k)=Z_{2}(k)-Z_{2}(k-1) \end{array}\right.$$

### 4.2. Proof of the Theorem

The above theorem can be proven as follows:

First, calculate the difference sequence (*i.e.*, the differences between every two adjacent measurements) of the two separate measurement systems:

(8) $$\begin{array}{l} \Delta Z_{1}(k)=Z_{1}(k)-Z_{1}(k-1)=\left[Z(k)-f_{1}(k)-V_{1}(k)\right]-\left[Z(k-1)-f_{1}(k-1)-V_{1}(k-1)\right]\\ \;=\left[Z(k)-Z(k-1)\right]+\left[f_{1}(k-1)-f_{1}(k)\right]+\left[V_{1}(k-1)-V_{1}(k)\right] \end{array}$$

(9) $$\begin{array}{l} \Delta Z_{2}(k)=Z_{2}(k)-Z_{2}(k-1)=\left[Z(k)-f_{2}(k)-V_{2}(k)\right]-\left[Z(k-1)-f_{2}(k-1)-V_{2}(k-1)\right]\\ \;=\left[Z(k)-Z(k-1)\right]+\left[f_{2}(k-1)-f_{2}(k)\right]+\left[V_{2}(k-1)-V_{2}(k)\right] \end{array}$$

Then, subtract the two difference sequences and yield the second order difference sequences; the trend items $f_{1}$ and $f_{2}$ are extremely small values compared to the measurement noise, so they are neglected after subtraction:

(10) $$\begin{array}{cc} \Delta Z_{1}(k)-\Delta Z_{2}(k) & =\left[f_{1}(k-1)-f_{1}(k)\right]-\left[f_{2}(k-1)-f_{2}(k)\right]\\ & +\left[V_{1}(k-1)-V_{1}(k)\right]-\left[V_{2}(k-1)-V_{2}(k)\right]\\ & \approx \left[V_{1}(k-1)-V_{1}(k)\right]-\left[V_{2}(k-1)-V_{2}(k)\right] \end{array}$$

Since $V_{1}(k)$ and $V_{2}(k)$ are uncorrelated, zero mean white noises, the autocorrelation of the second order difference sequences is:

(11) $$\begin{array}{c} E\left\{\left[\Delta Z_{1}(k)-\Delta Z_{2}(k)\right]{\left[\Delta Z_{1}(k)-\Delta Z_{2}(k)\right]}^{T}\right\}\\ =E\{\{\left[V_{1}(k-1)-V_{1}(k)\right]-\left[V_{2}(k-1)-V_{2}(k)\right]\}\times {\left\{\left[V_{1}(k-1)-V_{1}(k)\right]-\left[V_{2}(k-1)-V_{2}(k)\right]\right\}}^{T}\}\\ =E\{V_{1}(k-1)V_{1}^{T}(k-1)\}+E\{V_{1}(k)V_{1}^{T}(k)\}+E\{V_{2}(k-1)V_{2}^{T}(k-1)\}+E\{V_{2}(k)V_{2}^{T}(k)\} \end{array}$$

When the prerequisite Equation (5) is satisfied, the variance of measurement $Z_{1}$ can be calculated as:

(12) $$R_{1}=E\left[V_{1}(k)V_{1}{\left(k\right)}^{T}\right]\approx E\left\{\left[\Delta Z_{1}(k)-\Delta Z_{2}(k)\right]{\left[\Delta Z_{1}(k)-\Delta Z_{2}(k)\right]}^{T}\right\}/2$$

### 4.3. Availability of Using the Theorem in a GNSS/INS Tightly-Coupled System

The precondition of the theorem is that two separate measurement systems are available for the same value *Z*. This is suitable for the tightly-coupled integration system because the GNSS can provide the measurements of pseudo-range and pseudo-rate in a direct manner, and the INS can provide them in an indirect approach. Hence, the GNSS and INS are treated as Systems 1 and 2, respectively, in the proposed system.

On the other side, as the INS owns the short-term accuracy characteristic, the INS errors that accumulated in several seconds are much smaller than the GNSS errors and, thus, can be neglected. Therefore, the tightly-coupled GNSS/INS also meets the prior condition in Equation (5), that is:

(13) $$\left\{ \begin{array}{l} diag\left[\begin{array}{ccc} \cdots & {\left(f_{INS}^{i}(k)-f_{INS}^{i}(k-1)\right)}^{2} & \cdots \end{array}\right]\ll E[V_{GPS}(k)V_{GPS}{\left(k\right)}^{T}]\\ diag\left[\begin{array}{ccc} \cdots & {\left(f_{GPS}^{i}(k)-f_{GPS}^{i}(k-1)\right)}^{2} & \cdots \end{array}\right]\ll E[V_{GPS}(k)V_{GPS}{\left(k\right)}^{T}]\\ E[INS_{error}(k)INS_{error}{\left(k\right)}^{T}]\ll E[V_{GPS}(k)V_{GPS}{\left(k\right)}^{T}] \end{array}\right.$$

Hence, the proposed method can be applied in the tightly-coupled GNSS/INS system to estimate the variances of the GNSS pseudo-range and pseudo-rate noises. Furthermore, a sliding window strategy is designed for noise estimation. There are two main reasons for this design. First, the measurement noise is not always identically distributed and may change during the process; thus, using a sliding window can track the real-time noises accurately and mitigate the influence of historical information. Second, the INS errors are relatively smaller than the GNSS errors in each sliding window, which can lead to more accurate *R* estimation results. The formula for the second reason is:

(14) $$R_{1}=E\left[V_{1}(k)V_{1}{\left(k\right)}^{T}\right]\approx E\left\{\left[\Delta Z_{1}{\left(k\right)}_{k-M:k}-\Delta Z_{2}{\left(k\right)}_{k-M:k}\right]{\left[\Delta Z_{1}{\left(k\right)}_{k-M:k}-\Delta Z_{2}{\left(k\right)}_{k-M:k}\right]}^{T}\right\}/2$$

where *k* denotes the current time epoch and *M* denotes the size of the sliding window and is usually set as 20–50. The value of *M* decides the contribution of historical data to the *R* estimation.

## 5. Height/Heading-Aiding Modes

**Figure 3:**
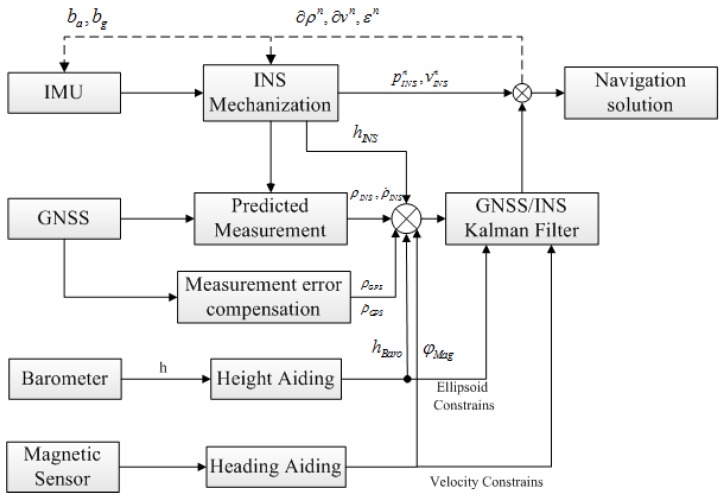
External measurement aiding system scheme.

Except for the adaptive Kalman filter, another important and indispensable measure is to introduce the external measurement, actually the height and heading aiding to improve the system performance in GNSS signal-challenged environment. The proposed ATCA system includes both the height and heading aiding, and the system block diagram is shown in Figure 3.

Figure 3 shows the height and heading-aiding mode in the tightly-coupled integration system. In order to effectively make use of these measurements and to maximize their contributions to improve the performance, this external information works in both directly aiding and measurement aiding manners in the proposed system, and the specific descriptions are provided in this section.

### 5.1. Height Aiding

The basic idea of using the height aiding is that if the height is constrained to a known value, then the remaining unknowns can be solved with one less measurement from the satellites. The height aiding is divided into two manners. The first one is the directly aiding manner, and it adds the height difference in the measurement model, which is directly relative to the state vector δh. The second one is the pseudo-measurement manner, and it assumes that the Earth is a static satellite, where an extra pseudo-range measurement is established and is added to the filter. In this way, it is able to provide a better position solution and to increase the horizontal solution.

#### 5.1.1. Direct Height Aiding

The difference between the height from external sources, such as a barometer, and the INS-computed height is added into the measurement equation. The barometer can measure the local atmospheric pressure and calculate the height to aid. Considering both GNSS and height updates, the measurement model under the height-aiding mode is:

(15) $$Z=\left[Z_{H}\right]=\left[H_{AUX}-H_{INS}\right]=H_{H}X(t)+V(t)$$

where $Z_{H}$ is the height difference, $H_{AUX}$ and $H_{INS}$ denote the height from auxiliary sensor and the INS derived result, measurement matrix is $H_{H}=\left[0_{1\times 8}\;1\;0_{1\times 8}\right]$ and V is the measurement noise.

In general, direct height aiding can enhance navigation performance by directly correcting for the height error and indirectly improving other states related to the height in the Kalman filter system model. To provide a stronger correlation between height measurement and the navigation states, a pseudo-measurement height aiding method is presented in the next subsection.

#### 5.1.2. Pseudo-Measurement Height Aiding

**Figure 4:**
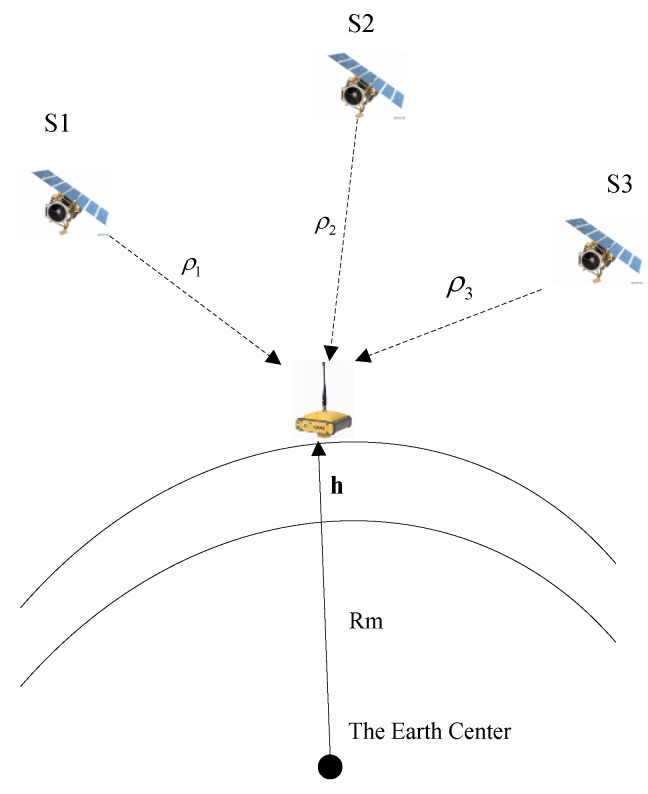
Height aiding scheme.

As shown in Figure 4, the Earth can be imaged as a pseudo-satellite, which keeps static at the Earth’s center (*i.e.*, the position of this pseudo-satellite is (0, 0, 0)). Therefore, an extra pseudo-satellite update can be applied, and its constraint the point lies on the surface of approximation ellipsoid.

Assuming the Earth’s surface as a reference ellipsoid, an ellipsoid equation can be employed to express the altitude constraint on the INS-derived position solution:

(16) $$\frac{{x_{INS}}^{2}}{{\left(R_{e}+h\right)}^{2}}+\frac{{y_{INS}}^{2}}{{\left(R_{e}+h\right)}^{2}}+\frac{{z_{INS}}^{2}}{{\left(R_{p}+h\right)}^{2}}=l_{1}$$

where $\left(x_{INS},y_{INS},z_{INS}\right)$ is the INS-derived position, $R_{e},R_{p}$ denote the length of the Earth’s semi-major and semi-minor axes, h is the altitude with respect to the Earth’s surface and $l_{1}$ is defined as the altitude constraint. The value of $l_{1}$ varies around one, and the difference between $l_{1}$ and 1 depends on the position error (δx,δy,δz). Linearizing Equation (16) around the true position (x,y,z) and ignoring the higher-order small items, we could obtain:

(17) $$\begin{array}{l} l_{1}=\frac{{x_{INS}}^{2}}{{\left(R_{e}+h\right)}^{2}}+\frac{{y_{INS}}^{2}}{{\left(R_{e}+h\right)}^{2}}+\frac{{z_{INS}}^{2}}{{\left(R_{p}+h\right)}^{2}}\\ \;=\frac{x^{2}}{{\left(R_{e}+h\right)}^{2}}+\frac{y^{2}}{{\left(R_{e}+h\right)}^{2}}+\frac{z^{2}}{{\left(R_{p}+h\right)}^{2}}+\frac{2x\delta x}{{\left(R_{e}+h\right)}^{2}}+\frac{2y\delta y}{{\left(R_{e}+h\right)}^{2}}+\frac{2z\delta z}{{\left(R_{p}+h\right)}^{2}}\\ \Delta l=l_{1}-1=\frac{2x\delta x}{{\left(R_{e}+h\right)}^{2}}+\frac{2y\delta y}{{\left(R_{e}+h\right)}^{2}}+\frac{2z\delta z}{{\left(R_{p}+h\right)}^{2}} \end{array}$$

Then the pseudo-measurement model could be established as:

(18) $$Z_{h}(t)=[l_{1}-1]=H_{h}X(t)+V(t)$$

where $H_{h}=[0_{1\times 6}\;h_{1}\;h_{2}\;h_{3}\;0_{1\times 8}]$, and:

(19) $$\begin{array}{l} h_{1}=\frac{-2x\left(R_{n}+h\right)\sin L\cos\lambda }{{\left(R_{e}+h\right)}^{2}}-\frac{2y\left(R_{n}+h\right)\cos L\sin\lambda }{{\left(R_{e}+h\right)}^{2}}+\frac{2z\cos L\cos\lambda }{{\left(R_{p}+h\right)}^{2}}\\ h_{2}=\frac{-2x\left(R_{n}+h\right)\sin L\sin\lambda }{{\left(R_{e}+h\right)}^{2}}+\frac{2y\left(R_{n}+h\right)\cos L\cos\lambda }{{\left(R_{e}+h\right)}^{2}}+\frac{2z\cos L\sin\lambda }{{\left(R_{p}+h\right)}^{2}}\\ h_{3}=\frac{2x[R_{n}(1-e^{2})+h]\cos L}{{\left(R_{e}+h\right)}^{2}}+\frac{2z\sin L}{{\left(R_{p}+h\right)}^{2}} \end{array}$$

Compared to the directly aiding manner, the pseudo measurement aiding approach can deeply relate the height measurements with the position solutions.

### 5.2. Heading Aiding

Typically, in heading aiding mode, we derive the external heading information with the magnetometer sensor and use it in two different manners to aid the ATCA system at the same time. The first one is the directly aiding manner, and it adds the heading difference in the measurement equation; the second one is the velocity measurement aiding manner, and it is based on the relationship between heading angle and horizontal velocities; here, the advantage of this manner is that it can provide correction for the velocities. In the proposed heading aiding mode, we implicitly assume that the receiver is only moving in the direction of its heading, and it will not move in the side direction or have other complex motion types. This actually is a kind of implicit NHC, but does not require all traditional NHC conditions, which is also true and can be applied in many practical cases.

Followed by the heading aiding manners, after the calibration of the magnetometer [31], we introduce how to derive an absolute heading through a commonly-used magnetometer sensor, and it follows these steps [32]: (1) leveling the magnetometer measurements by using roll and pitch angles; (2) using the leveled magnetometer measurements to calculate the magnetic heading (*i.e.*, the heading angle from the Earth’s magnetic north); and (3) calculating the true heading (*i.e.*, the heading angle from the Earth’s geographic north) by adding a declination angle to the magnetic heading.

#### 5.2.1. Direct Heading Aiding

In this aiding manner, the difference between the magnetometer heading and the INS-derived heading is added into the measurement model as:

(20) $$Z=\left[Z_{\varphi }(t)\right]=\left[\varphi_{mag}-\varphi_{INS}\right]=H_{\varphi }(t)X(t)+V(t)$$

where $Z_{\varphi }(t)$ is the added heading observation, $\varphi_{mag},\varphi_{INS}$ denote the heading from magnetometers and INS, respectively, $H_{\varphi }(t)=\left[0\;0\;1\;0_{1\times 14}\right]$ means the measurement matrix and V is measurement noise.

#### 5.2.2. Velocity Measurement Aiding

The derivation of this heading aiding manner starts from the relationship between heading and the horizontal velocities. The true heading φ and the INS-derived heading $\varphi_{INS}$ can be written as:

(21) $$\begin{array}{l} \;\varphi =\arctan\frac{V_{E}}{V_{N}}\;\\ \varphi_{INS}=\arctan\frac{V{’}_{E}}{V{’}_{N}}=\arctan\frac{\left(V_{E}+\delta V_{E}\right)}{\left(V_{N}+\delta V_{N}\right)} \end{array}$$

where $V_{N}$ and $V_{E}$ denote the velocity in north and east in the local level frame, $\delta V_{N}$ and $\delta V_{E}$ denote the velocity error. Linearizing the INS heading equation around $\left(V_{E},V_{N}\right)$ and keeping only first-order terms, the equation becomes:

(22) $$\varphi_{INS}=\arctan\frac{V_{E}}{V_{N}}+\frac{V_{E}\times V_{N}^{2}}{V_{E}^{2}+V_{N}^{2}}\delta V_{N}-\frac{1}{V_{E}^{2}+V_{N}^{2}}\delta V_{E}\;$$

Substituting Equation (21) into Equation (22), the heading error equation is:

(23) $$\varphi_{INS}-\varphi =\frac{V_{E}\times V_{N}^{2}}{V_{E}^{2}+V_{N}^{2}}\delta V_{N}-\frac{1}{V_{E}^{2}+V_{N}^{2}}\delta V_{E}$$

Then, the measurement model for the heading aiding is:

(24) $$Z(t)=\delta \varphi =[\varphi_{mag}-\varphi_{INS}]=H_{\varphi }X(t)+V(t)$$

where $H_{\varphi }=[0_{1\times 3}\;-\frac{1}{V_{E}^{2}+V_{N}^{2}}\;\frac{V_{E}\times V_{N}^{2}}{V_{E}^{2}+V_{N}^{2}}\;0_{1\times 12}]$, $\varphi_{mag}=\varphi +V_{mag}$, $V_{mag}$ denotes the measurement noise.

The measurement model also revealed that this external heading aiding contributes to the system when the vehicle’s speed is sufficiently high; otherwise, it may bring error to the velocity estimation. In this system, this heading aiding is activated only when the vehicle’s speed exceeds 5 m/s.

## 6. System Platform Implementation

We design and implement a tightly-coupled integration system to test the proposed method. The system is comprised of a low-cost IMU, a GNSS receiver, a magnetometer, a barometer and a core processor. The high performance digital signal processor (DSP) TMS320C6416 from Texas Instrument (Dallas, TX, USA) is chosen as the core processor for the proposed system, and the basic command operations, data collection, time synchronization and the navigation solutions’ calculation are all built-in. The processor is configured with 2 MB static RAM, 16 MB SDRAM and 4 MB flash memory, and the processing frequency is set up to 1 GHz, where the storage configuration and powerful processing ability guarantee that the proposed tightly-coupled algorithm is capable of running on the device in real time.

The device is configured with four UARTs for data collection with the TL16C752B chip from Texas Instrument (Dallas, TX, USA). The TL16C752B is a dual universal asynchronous receiver/transmitter (UART) with 64-byte FIFOs (First Input First Output); it also has the function of automatic hardware/software flow control, and the data rates can be up to 3 Mbps. Of all of the available four UARTs, three of them are for the INS, GNSS and magnetometer input; the last one is just for the navigation solution output and is connected to the user interface.

The GNSS pulse-per-second (PPS) signal is required for the time synchronization process and is connected to the external interrupt pin of the device. When the PPS signal is generated, it will trigger an interrupt in the processor, and the INS data and the auxiliary measurement data are all time-tagged with the GNSS time; they are in the same time frame [33].

**Figure 5:**
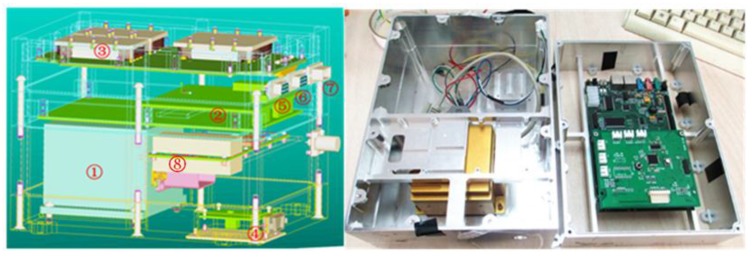
System constitution.

The system combination and constitution are shown in the Figure 5, and all of the components are listed in Table 1. All of the parts of the system are installed in a case and are connected by cables or jumpers inside the case.

**Table 1:** System components.

No.	Component	No.	Component
1	Crossbow IMU-440	5	Output Interface (RS-232)
2	Core Processor (6416)	6	JTAG (Joint Test Action Group) interface
3	Voltage Converter (28 V to 5 V)	7	Voltage Input (28 V)
4	Magnetometer TCM5	8	GNSS Receiver

The system is comprised of the Crossbow (Milpitas, CA, USA) IMU-440 MEMS sensor, a GNSS receiver, the TCM5 magnetometer by PNI Company (Santa Rosa, CA, USA) and the MS5803 low-cost barometer. The dimensions of the whole system are 238 mm × 172 mm × 200 mm (B × L × H), with a ±0.5 mm dimensional tolerances, and the system power consumption is 33.6 W, where the input voltage is 28 V and the measured current is 1.2 A. The Crossbow (Milpitas, CA, USA) IMU440 MEMS inertial measurement unit used in this system is a six DOF (degree of freedom) MEMS inertial sensor cluster that includes three axes of MEMS angular rate sensing and three axes of MEMS linear acceleration sensing. These sensors are based on rugged, field-proven silicon bulk micromachining technology [34]. The gyroscope’s bias stability is 10°/h; the angular random walk is 4.5 $\text{°/}\sqrt{\text{h}}$; and the measurement range is ±200°/s and can be set up to ±400°/s. The accelerometer’s bias stability is 1 mg; the velocity random walk is 0.5 $\text{m/s/}\sqrt{\text{h}}$; and the measurement range is ±4 g and can be set up to ±10 g.

The TCM5 is a low power, electronic tilt-compensated compass sensor module. It integrates a three-axis magnetic-field sensing with three-axis tilt sensing together and can provide the compass heading information [35]. The TCM5 is capable of providing pitch, roll and azimuth angle together, but only the azimuth output is used in the proposed system.

## 7. Tests and Results

We conduct both computer simulation and practical experiments to evaluate the effectiveness and performance of the proposed system, and they are illustrated in the following subsections. We utilized computer simulation to investigate the quality of the algorithm when less than four satellites are tracked and guide the design of real tests in advance. Furthermore, we conducted land-based vehicle driving tests for verification.

### 7.1. The Description of the Algorithms for Comparison

In order to have a further assessment of our proposed system, we also employ several previously existing methods here to calculate the navigation solution and compare all of the results together to evaluate the performance. The methods used for the comparison are briefly described below:

1. Standard tightly-coupled integrated system: also referred to as centralized integration. An integration filter is used to fuse INS and GPS measurement. The raw pseudo-range and Doppler measurements from GPS tracking loop output and those from INS prediction are combined to form the input of the centralized integration filter. The filter directly accepts their differences to obtain the INS error estimates [22]. This approach is represented as Standard TC in the following illustration.
2. Standard tightly-coupled integrated system with height and heading aiding: based on the standard tightly-coupled integration system, the external height and heading information are involved in the measurement model of the filter; the differences of INS-derived height and heading and the measured height and heading (from barometer and magnetometer) are added in the measurement equation for the update [23]. This approach is represented as TCA (tightly-coupled with height and heading aiding) in the following illustration.
3. Standard tightly-coupled integrated system with height and heading aiding and the improved Sage-Husa (SG) method for measurement noise estimation: An adaptive measurement noise estimation strategy using the improved SG method is introduced in the previously described “standard tightly-coupled integrated system with height and heading aiding” method. The improved SG is the most commonly-used noise estimation method in adaptive Kalman filter [28]; it is an innovation based adaptive estimation (IAE), which utilizes new statistical information from the innovation sequence to correct the estimation of the states. The measurement noise covariance is derived from the innovative sequence according to the following equation:
  (25) $$\begin{array}{l} d_{k}=z_{k}-H_{k}x_{k}^{-}\\ R_{k}=\frac{1}{N}\sum_{i=0}^{N-1}d_{k-i}d_{k-i}^{T} \end{array}$$
  where $z_{k}$, $H_{k}$ and $x_{k}^{-}$$H_{k}$ denote the measurement, measurement matrix and prediction in the Kalman filter. $d_{k}$ denotes the innovative sequence, N denotes the window size, and $R_{k}$ denotes the noise covariance. This approach is represented as TCA with SG in the following illustration.

### 7.2. Simulation Experiment

**Figure 6:**
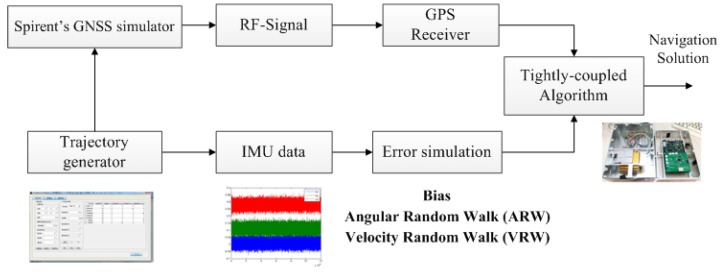
Simulation experiment scheme.

The benefit of simulation experiments is that the specific test scenarios can be created by software; therefore, it is feasible to obtain the true values to compare with system solutions for algorithm evaluation. The block diagram of the simulation process is shown in Figure 6. A trajectory generator was employed to produce the desired test trajectory and corresponding true IMU data. Then, errors, such as bias, random walk and scale factor errors, were added into the true IMU data to mimic the measured IMU data. Meanwhile, the generated trajectory file was imported into the Spirent GNSS simulator software suite SimGEN™ (Spirent Company, Sunnyvale, CA, USA) to simulate the GNSS data. Finally, the GNSS output was collected and integrated with IMU data for the navigation solution.

The simulated trajectory is shown in Figure 6. This trail consisted of three stages, including linear motion with a constant acceleration, uniform linear motion and turning with a constant angular velocity. The satellite visible number was adjusted to less than four randomly in the navigation process through the SimGEN ™ software by turning off some satellite channels. The simulated gyroscope biases were set as 10°/h, and the angular random walk was 20 $\text{°/}\sqrt{\text{h}}$, while the accelerometer biases and velocity random walk were set as 1 mg and 1 $\text{m/s/}\sqrt{\text{h}}$, respectively. Meanwhile, the simulated height and heading were generated by adding Gaussian distributed noises to the true values.

**Figure 7:**
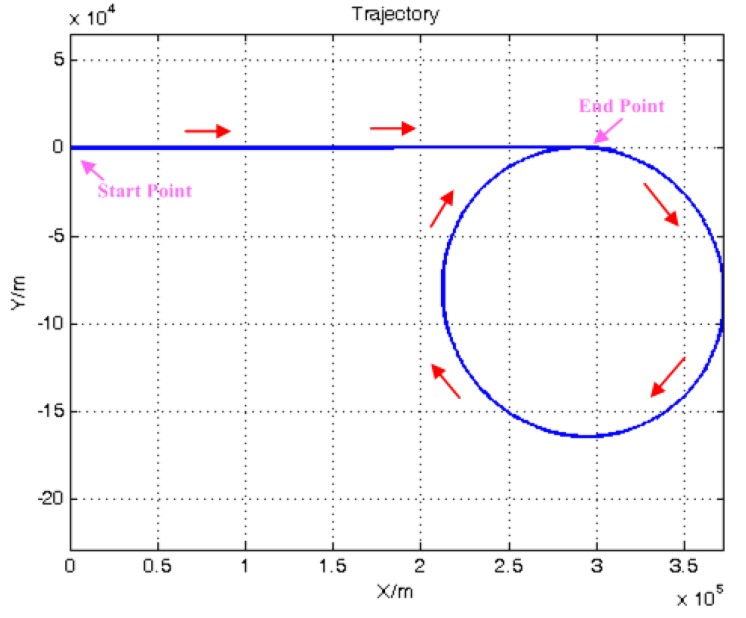
Simulated trajectory.

Figure 7 shows the flight trajectory, and the red arrows show the flight directions. The trajectory is shown in meters scale, and the start point is set as (0, 0). The simulated data are processed with the standard TC, TCA, TCA with SG and ATCA previously discussed for performance evaluation. The initial values of the filters are set the same to assess the navigation solution in the same situation, and the parameters configurations are listed as follows:

(26) $$\begin{array}{l} Q=diag\{{\left(1\;\text{m/s/}\sqrt{\text{h}}\right)}^{2},\;{\left(1\;\text{m/s/}\sqrt{\text{h}}\right)}^{2},{\left(1\;\text{m/s/}\sqrt{\text{h}}\right)}^{2},{\left(20\;{}^{\circ}/\sqrt{h}\right)}^{2},{\left(20\;{}^{\circ}/\sqrt{h}\right)}^{2},{\left(20\;{}^{\circ}/\sqrt{h}\right)}^{2}\\ \;{\left(1{}^{\circ}/h\right)}^{2},\;{\left(1{}^{\circ}/h\right)}^{2},\;{\left(1{}^{\circ}/h\right)}^{2},\;{\left(1\;\text{mg}\right)}^{2},\;{\left(1\;\text{mg}\right)}^{2},{\left(1\;\text{mg}\right)}^{2},{\left(1\;\text{m}\right)}^{2},{\left(0.1\;\text{m/s}\right)}^{2}\}\\ R=diag\{1^{2},\;1^{2},\;1^{2},\;1^{2},\;0.1^{2},\;0.1^{2},\;0.1^{2},\;0.1^{2}\}\\ X=\left[0\;0\;0\;0\;0\;0\;0\;0\;0\;1\;{}^{\circ}\text{/}h\;1\;{}^{\circ}\text{/}h\;1\;{}^{\circ}\text{/}h\;1\;\text{mg }1\;\text{mg }1\;\text{mg 1}\;\text{m 0.1m/s}\right]\\ P=X^{T}X \end{array}$$

**Figure 8:**
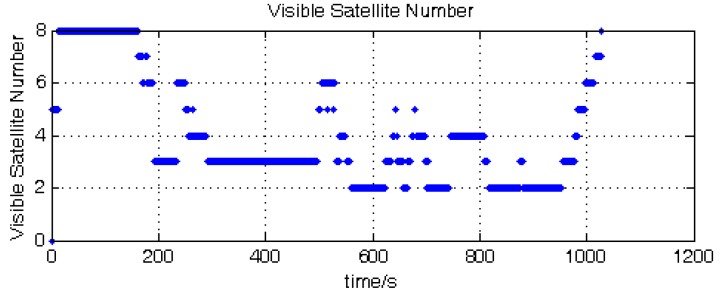
Satellite visible number.

**Figure 9:**
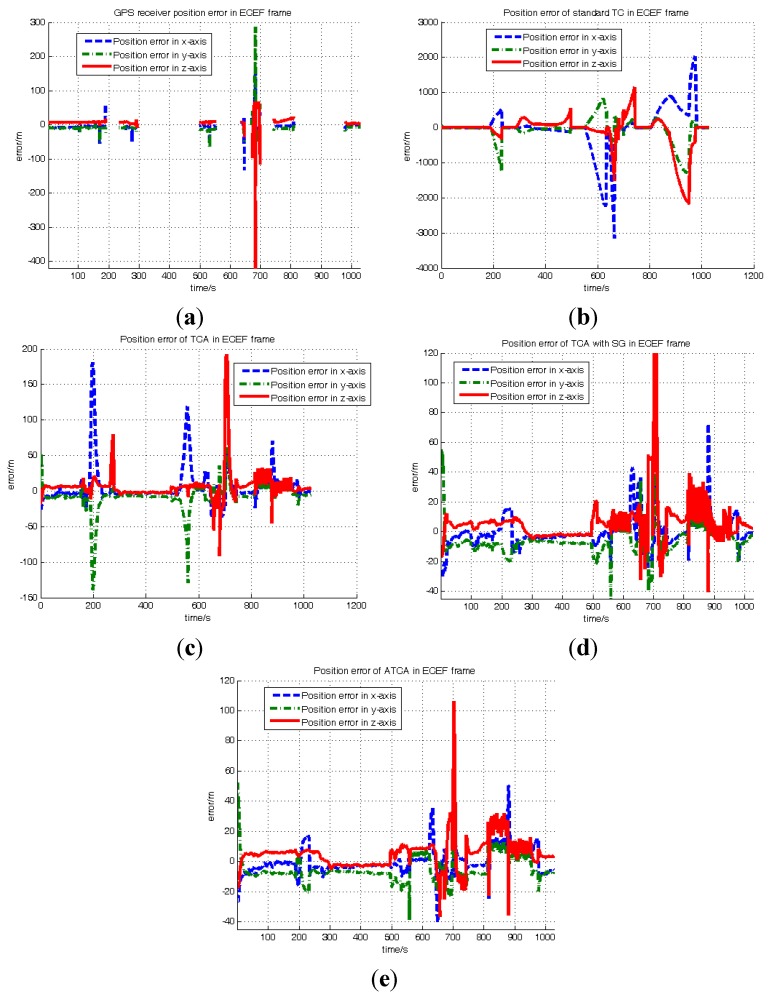
Position errors of different methods: (**a**) GNSS receiver error; (**b**) standard TC position error; (**c**) TCA position error; (**d**) TCA with SG position error; (**e**) ATCA position error.

The simulation experiment lasted 1020 s, and during this process, the visible satellite number is randomly changed through the SimGen™ software. The satellite visible number is shown in Figure 8. The position results of GNSS receiver and the four compared tightly-coupled algorithms are shown in Figure 9.

Figure 9 shows the position error in the ECEF (Earth-Centered, Earth-Fixed) frame of the GNSS receiver and the four compared tight-coupled methods. Figure 9a denotes the GNSS receiver position error; it can be seen that the receiver has no position output in some periods, because the satellite number is below four.

Figure 9b–e denotes the position errors of standard TC, TCA, TCA with SG and ATCA. Table 2 lists the RMS position error results of the GNSS receiver and the compared tightly-coupled algorithms, where it includes the periods that the satellite visible number is more than four and less than four in the whole experiment. It is obvious that the proposed ATCA algorithm has the least position error overall no matter the satellite visible number being more than four or less than four. In order to further evaluate the proposed method, we also selected four periods in this experiment to compare the position error and to see more details.

**Table 2:** RMS position error result.

	Satellite Number More than 4			Satellite Number Less than 4		
	X (m)	Y (m)	Z (m)	X (m)	Y (m)	Z (m)
GNSS receiver	12.1204	19.7539	26.4187	NA	NA	NA
Standard TC	10.4966	14.4910	24.6421	778.5215	456.6524	643.8866
TCA	10.0638	9.7813	13.5467	33.3155	27.1117	26.2960
TCA with SG	7.9877	11.8187	11.7559	13.0102	11.0251	24.4045
ATCA	5.4093	9.6268	8.2195	10.0279	8.8454	13.3769

**Figure 10:**
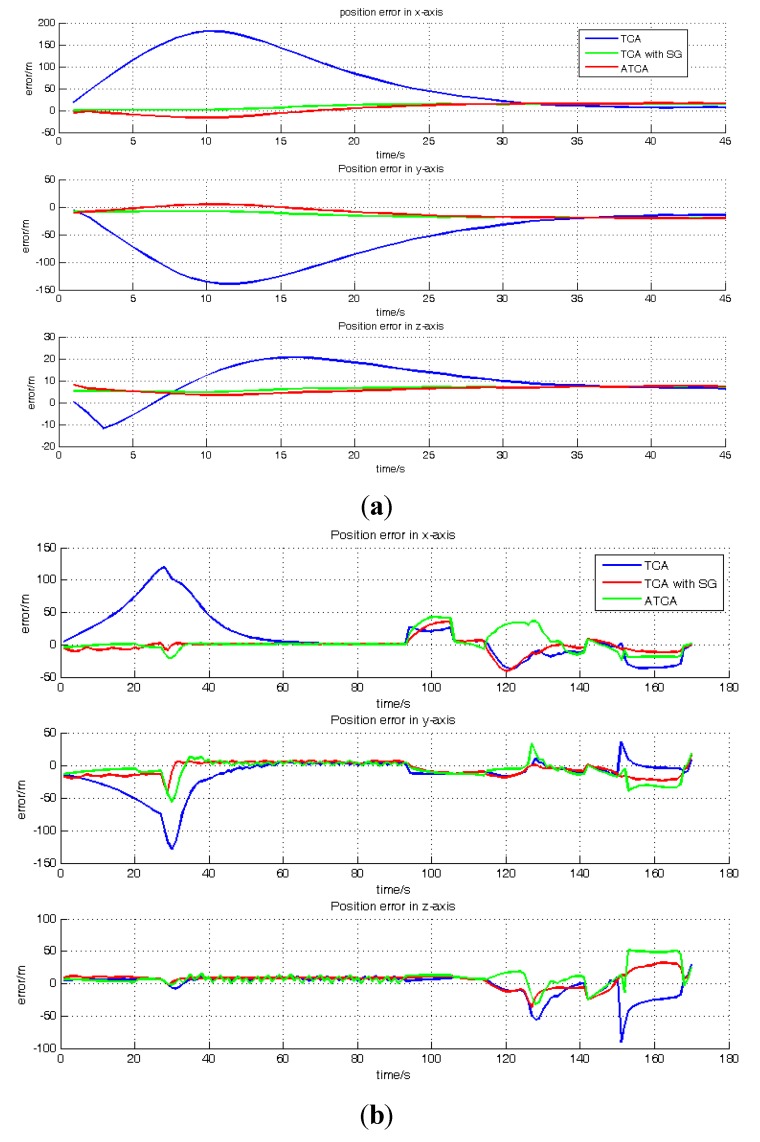
Position error comparisons: (**a**) position errors of TCA, TCA with SG and ATCA in the ECEF (Earth-Centered, Earth-Fixed) frame during the periods of 188 s and 233 s; (**b**) position errors of TCA, TCA with SG and ATCA in the ECEF frame during the periods of 531 s and 700 s.

Figure 10a,b shows the position error results of TCA, TCA with SG and ATCA during the periods of 189–233 s and 531–700 s. Table 3 shows the RMS of position error in these two periods. Because the satellite visible number is less than four in these two periods, the GNSS receiver has no output, and the performance of the standard TC is much worse than the other three methods. Hence, for a better comparison and presentation in the figure, only the position results of TCA, TCA with SG and ATCA are shown in the figures, but the standard TC position error result is listed in Table 3.

**Table 3:** RMS position error results.

	The Period of 189–233 s			The Period of 531–700 s		
	X (m)	Y (m)	Z (m)	X (m)	Y (m)	Z (m)
Standard TC	350.6096	707.5608	198.8642	1163.5	367.2	342.6
TCA	93.3646	77.1261	12.6208	37.7900	28.5764	16.7951
TCA with SG	11.5668	15.5311	6.4813	15.2633	14.6680	17.4740
ATCA	13.0553	14.0751	6.0460	12.0622	12.1148	12.9468

**Figure 11:**
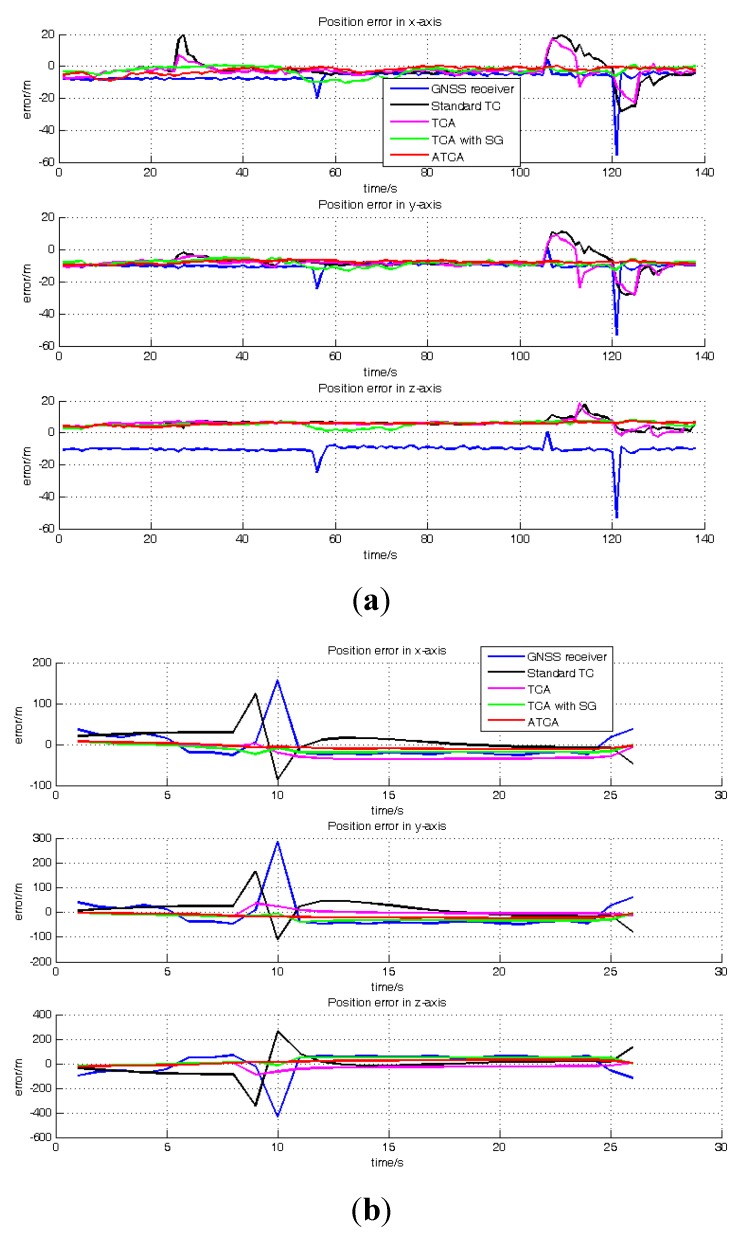
Position error comparisons: (**a**) position errors of GNSS receiver, standard TC, TCA, TCA with SG and ATCA in the ECEF frame during the periods of 50 s and 187 s; (**b**) position errors of the GNSS receiver, standard TC, TCA and ATCA in the ECEF frame during the periods of 673 s and 698 s.

Figure 11a and b shows the position result of the GNSS receiver, standard TC, TCA, TCA with SG, ATCA during the periods of 50–187 s and 673–698 s. They are presented in the blue line, black line, pink line, green line and red line. Table 4 lists the RMS of the position error in these two periods. The visible satellite number during the two periods is always more than four, and the GNSS receiver works normally in the two periods.

Based on the position performance comparisons of several selected periods and the overall results, we can conclude that the proposed ATCA method has the least error and is capable of providing the best navigation solutions in the simulation experiment.

**Table 4:** RMS position errors.

	The Period of 50–187 s			The Period of 673–698 s		
	X (m)	Y (m)	Z (m)	X (m)	Y (m)	Z (m)
GNSS	8.0610	11.2364	7.0904	38.1481	67.8798	104.6477
Standard TC	7.6642	9.8481	6.3391	34.9907	47.0839	99.4847
TCA	5.9293	10.1106	6.1428	26.5837	10.8538	31.2858
TCA with SG	4.1211	8.9007	5.4018	15.4418	26.3500	38.1509
ATCA	3.1442	8.0449	5.7103	8.7274	17.6114	22.9864

### 7.3. Practical Experiment

In the practical experiment, post-mission processing using the real-time algorithms is used to assess the integration system performance. Though the algorithm was demonstrated in post-processing mode, no special pre-processing of the data was required. A series of tests were conducted to verify the performance of the approach proposed in this paper.

The initial values and the parameter configuration of the filters for the compared methods are listed as follows:

$$\begin{array}{l} Q=diag\{{\left(0.5\text{m/s/}\sqrt{\text{h}}\right)}^{2},\;{\left(0.5\;\text{m/s/}\sqrt{\text{h}}\right)}^{2},{\left(0.5\;\text{m/s/}\sqrt{\text{h}}\right)}^{2},{\left(4.5\;{}^{\circ}/\sqrt{h}\right)}^{2},{\left(4.5\;{}^{\circ}/\sqrt{h}\right)}^{2},{\left(4.5\;{}^{\circ}/\sqrt{h}\right)}^{2}\\ \;{\left(1{}^{\circ}/h\right)}^{2},\;{\left(1{}^{\circ}/h\right)}^{2},\;{\left(1{}^{\circ}/h\right)}^{2},\;{\left(1\;\text{mg}\right)}^{2},\;{\left(1\;\text{mg}\right)}^{2},{\left(1\;\text{mg}\right)}^{2},{\left(1\;\text{m}\right)}^{2},{\left(0.1\;\text{m/s}\right)}^{2}\}\\ R=diag\{1^{2},\;1^{2},\;1^{2},\;1^{2},\;0.1^{2},\;0.1^{2},\;0.1^{2},\;0.1^{2}\}\\ X=\left[0\;0\;0\;0\;0\;0\;0\;0\;0\;1\;{}^{\circ}\text{/}h\;1\;{}^{\circ}\text{/}h\;1\;{}^{\circ}\text{/}h\;1\;\text{mg }1\;\text{mg }1\;\text{mg 1}\;\text{m 0.1m/s}\right]\\ P=X^{T}X \end{array}$$

The field test was performed in Beijing, China, and the driving route is around the Beijing National Stadium. The IMU, GNSS receiver and the auxiliary measurement equipment are mounted in the vehicle. The inertial data are collected by the Crossbow (Milpitas, CA, USA) MEMS IMU 440, and the barometer MS5083 and magnetometer TCM5 are used as the auxiliary sensors to measure the height and magnetic heading.

**Figure 12:**
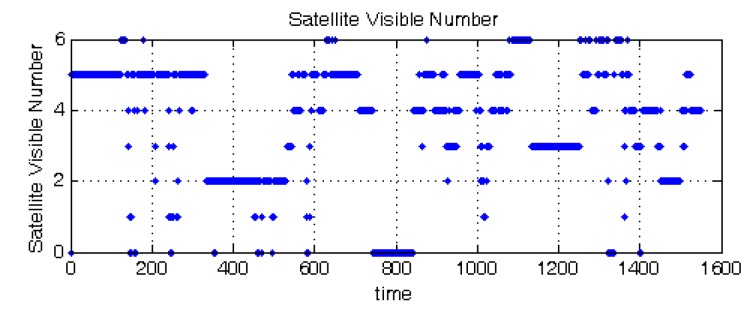
Satellite visible number in the practical experiment.

The whole test lasted about half an hour, and the data were collected for verification. Both “simulated” outages and “real” partial outages existed in the processed data. The simulated outage means that if the satellite visible number is more than two or three in this period, this is treated as two or three satellites being tracked, and randomly, two or three satellites’ information is used for the calculation. In this field test, both the 345–530-s and 1450–1500-s periods are simulated as the two visible satellites situation, and the 1130–1300-s period is simulated as the three visible satellites situation. The satellite visible number during the process is shown in Figure 12.

**Figure 13:**
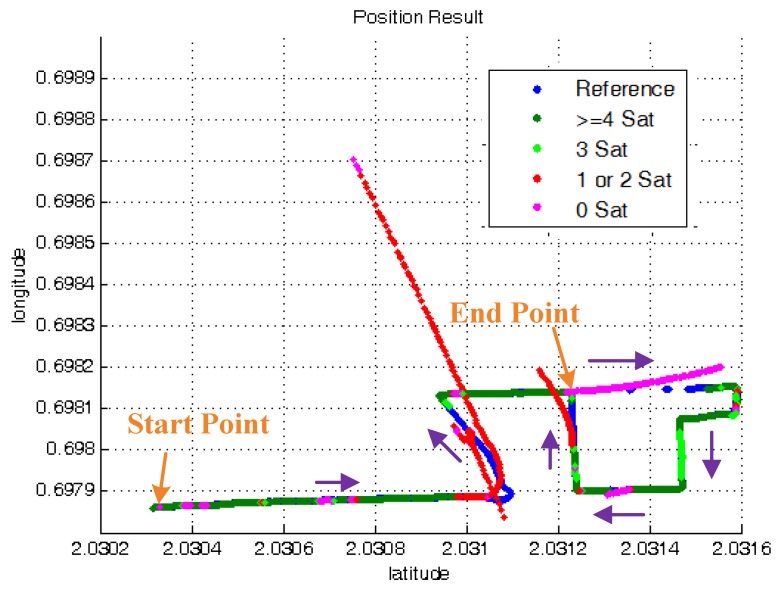
Standard tightly-coupled position result.

Figure 13 shows the position result of standard tight-coupled solution for the practical experiment. The blue points denote the reference trajectory. The dark green points denote the tightly-coupled solution with more than four satellites. The light green points denote the solution with three satellites. The red and pink points separately denote the position result with two satellites and less than two satellites. It can be seen in the figure that during the two visible satellites periods, the result of the standard tightly-coupled method drifts from the true trajectory greatly and has a large position error.

**Figure 14:**
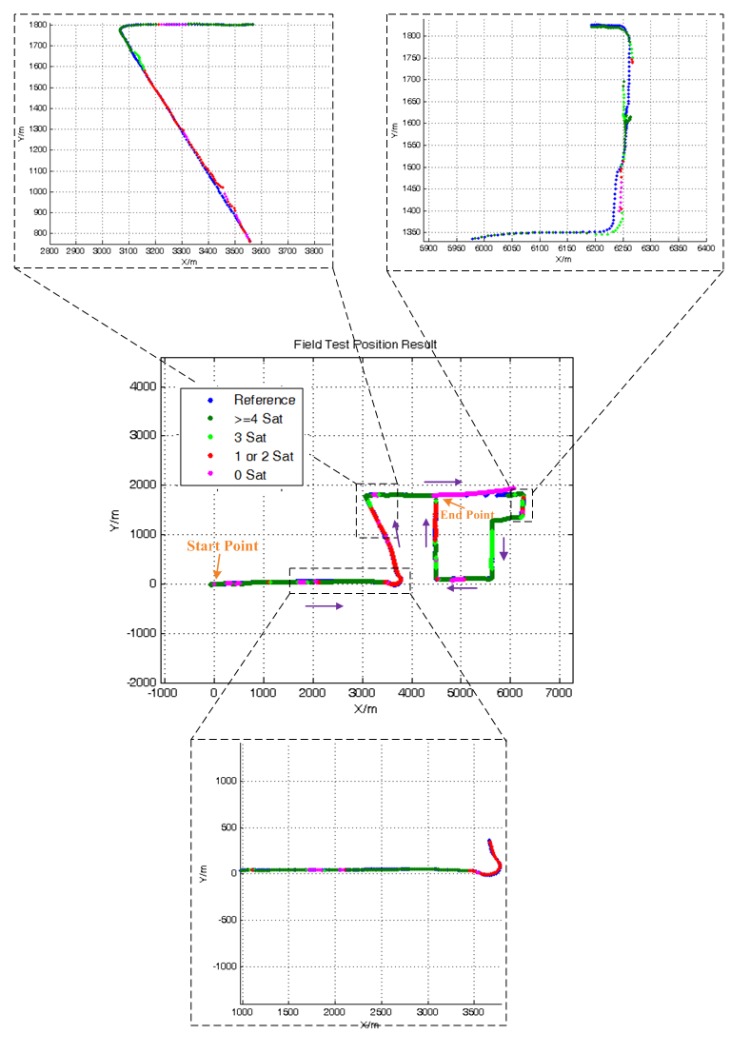
Position result of ATCA.

Figure 14 shows the whole trajectory result of ATCA and some zoomed details. The result obviously shows that the proposed method is also capable of solving the navigation problem in a GNSS signal-challenged environment in practice and is much better than the standard TC.

The TCA and TCA with SG are also implemented for evaluation and comparison. The differences between TCA, TCA with SG and ATCA are hard to identify, so the TCA result is not shown in the figures. However, the RMS of the position error is listed in Table 5 for comparison. Moreover, we also select two periods where only two satellites are visible to compare the solution.

**Figure 15:**
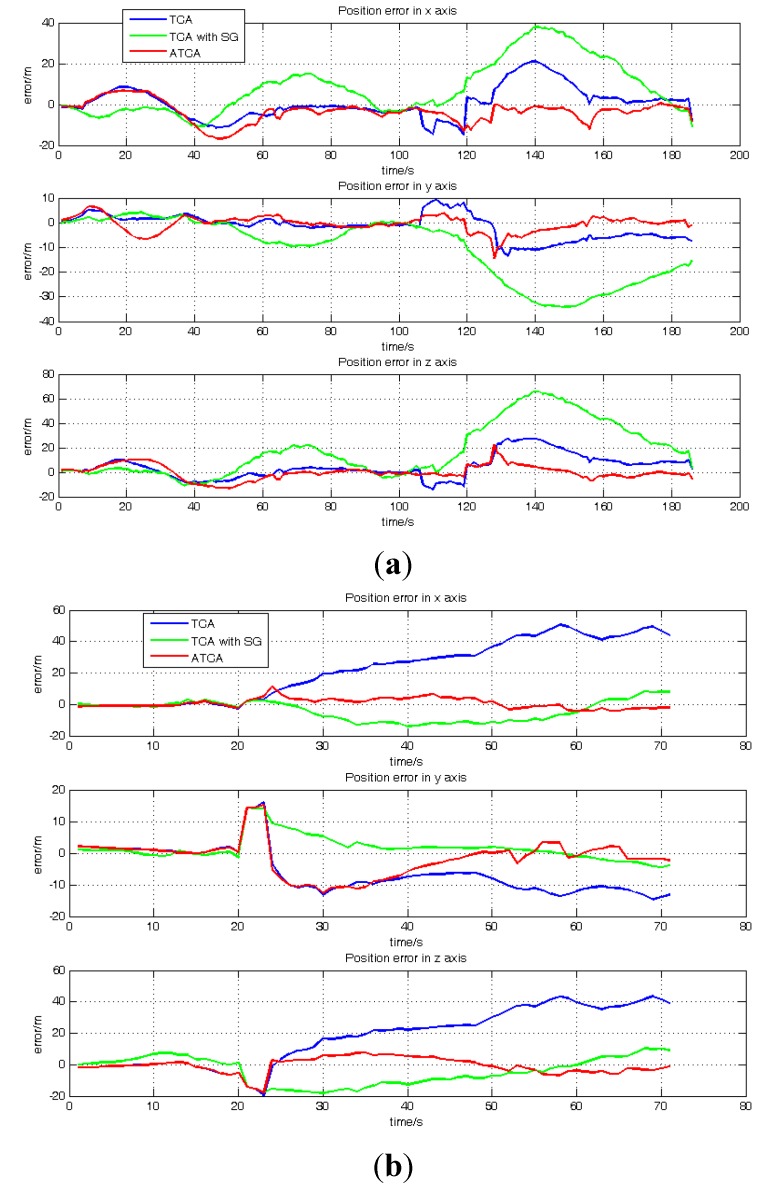
Position error comparisons: (**a**) position errors of TCA, TCA with SG and ATCA in the ECEF frame during the periods of 345 s and 530 s; (**b**) position errors of TCA, TCA with SG and ATCA in the ECEF frame during the periods of 1450 s and 1500 s.

Table 5 lists the RMS of the position error for the whole practical experiment, and it shows that the ATCA has the least position error. Figure 15 shows the position errors of TCA, TCA with SG and ATCA in the two simulated two visible satellites periods, and Table 6 lists the RMS of position errors. The practical results also show that the proposed ATCA has the best performance and can provide the best position solutions with less than four visible satellites.

After both the simulation experiment and practical land vehicle test, the navigation solution results of standard TC, TCA, TCA with SG and ATCA have been described in the figures and listed in the tables. The specific analyses of the four compared methods are illustrated in the following.

The standard TC obviously diverges and suffers from the largest position errors, which can be up to hundreds of meters; the reason for this is that the system becomes unobservable because of the lack of enough measurement for the position solution.

The TCA has a much better performance compared to the standard TC. The position errors of TCA become convergent and can be constrained to less than 100 m; this is because the external height and heading information improves the position constraints of the whole system, and the system becomes observable. However, in some periods, the position error will increase to 20–50 m and slightly decrease to a normal range, as shown in Figure 10; this is because at several points, the system measurements (pseudo-range and pseudo-rate) suffer from a large measuring noise because of signal blockage; the filter still makes use of the constant measurement covariance model and leads to the error in the final solution.

**Table 5:** RMS of the position error in the ECEF frame of the whole experiment.

	Error X (m)	Error Y (m)	Error Z (m)
Standard TC	355.8420	276.7677	483.7556
TCA	8.4694	4.0710	8.1874
TCA with SG	6.4785	3.8244	8.8093
ATCA	3.4617	3.6882	4.3391

**Table 6:** RMS position error in the two periods.

	The Period of 345–530 s			The Period of 1450–1500 s		
	X (m)	Y (m)	Z (m)	X (m)	Y (m)	Z (m)
Standard TC	794.757	628.0711	1076.4786	126.3409	39.7067	109.4160
TCA	7.7362	4.85646	9.48123	18.6519	6.94387	17.7130
TCA with SG	13.1257	12.4301	21.6142	6.4785	3.8244	8.8093
ATCA	5.1612	3.14215	5.86392	3.17873	5.62555	5.11066

The TCA with SG involves the SG method in TCA and employs the innovation sequence to adaptively estimate and tune the measurement noise covariance *R* online. With this strategy, the measurement quality can be evaluated, and the noise covariance is adjusted based on the actual situation instead of employing a constant value. The results listed in the Table 2, Table 3 and Table 5 have demonstrated that the TCA with SG successfully avoid the position error appeared in TCA, which is caused by the signal blockage. As illustrated in Figure 11b and Figure 15a, in some periods, the TCA with SG performs worse than TCA and suffers from a larger position error, the reason for this is that the system state vector *X* is not well estimated, and it affects the estimation of covariance noise *R* in these two durations. However, though the TCA with SG cannot perform well all of the time and have larger position error in some periods, this method still owns an overall better performance than TCA.

The ATCA has the best performance of all. This proposed method is able to effectively make use of external measurement height and heading together and add them in the measurement mode to increase the system observability. On the other side, it introduces the redundant measurement noise estimation method to adaptively tune the *R* online to avoid the negative effect of GNSS measurement noise. Compared to TCA with SG, which also owns the adaptively tuning strategy to estimate the measurement noise, the novel proposed redundant measurement noise estimation method used in ATCA, which utilizes the second order difference sequence to estimate the noise variance, totally relies on the measurement system itself to acquire the noise information without coupling the system state error. However, the Sage-Husa method is based on the innovation sequence, and the database for noise estimation is the difference of measurement *Z* and system prediction *X^−^*, which means that the estimation error of *X* will be involved in the noise estimation to affect the filter. Hence, the ATCA performs better, has less position errors than TCA with SG and has the best solution result of all of the compared methods.

## 8. Conclusions

In this paper, we put forward a novel adaptive low-cost GNSS/MEMS-IMU tightly-coupled integration system that can provide satisfactory navigation solutions in a GNSS signal-challenged environment when less than four satellites are visible. The proposed system features an adaptive measurement noise estimation method, which is totally based on the measurement system and decoupled from state vector error. Moreover, we also design a tightly-coupled integration manner of contributing the external measurement height and heading angle to the filter. The hardware platform of the proposed system is established by combining the DSP processor, GPS receiver, IMU, barometer and magnetometer. Both simulation and practical experiments were conducted to test and verify the system. The results show that the proposed ATCA is capable of offering seamless navigation solutions in harsh environments and has the best performance compared to the standard tight-coupled system and the tightly-coupled system with aiding measurement.

## Appendix

### A. System State Mode

As described in Equation (2), the system state matrix is the combination of three non-zero matrices ${\left(F_{I}\right)}_{9\times 9}$, ${\left(F_{M}\right)}_{6\times 6}$, ${\left(F_{G}\right)}_{2\times 2}$ and $\Gamma_{I}(t)$. The specific components of the three matrices are listed below.

#### A.1. First Matrix ${\left(F_{I}\right)}_{9\times 9}$

$F(1,2)=w_{ie}\sin L+\frac{v_{e}}{R_{n}+h}\tan L$	$F(1,3)=-w_{ie}\cos L-\frac{v_{e}}{R_{n}+h}$	
$F(1,5)=-\frac{1}{R_{m}+h}$	$F(2,1)=-w_{ie}\sin L-\frac{v_{e}}{R_{n}+h}\tan L$	
$F(2,3)=-\frac{v_{n}}{R_{m}+h}$	$F(2,4)=\frac{1}{R_{n}+h}$	
$F(2,7)=-w_{ie}\sin L$	$F(3,1)=w_{ie}\cos L+\frac{v_{e}}{R_{n}+h}$	
$F(3,2)=\frac{v_{n}}{R_{m}+h}$	$F(3,4)=\frac{1}{R_{n}+h}\tan L$	
$F(3,7)=w_{ie}\cos L+\frac{v_{e}}{R_{n}+h}\sec^{2}L$	$F(4,2)=-f_{u}F(4,3)=f_{n}$	
$F(4,4)=\frac{v_{n}}{R_{n}+h}\tan L-\frac{v_{u}}{R_{n}+h}$	$F(4,5)=2w_{ie}\sin L+\frac{v_{e}}{R_{n}+h}\tan L$	
$F(4,6)=-2w_{ie}\cos L-\frac{v_{e}}{R_{n}+h}$	$F(4,7)=2w_{ie}v_{n}\cos L+\frac{v_{e}v_{n}}{R_{n}+h}\sec^{2}L+2w_{ie}v_{u}\sin L$	
$F(5,1)=f_{u}F(5,3)=-f_{e}$	$F(5,4)=-2(w_{ie}\sin L+\frac{v_{e}}{R_{n}+h}\tan L)$	
$F(5,5)=-\frac{v_{u}}{R_{m}+h}$	$F(5,6)=-\frac{v_{n}}{R_{m}+h}$	
$F(5,7)=-(2w_{ie}\cos L+\frac{v_{e}}{R_{n}+h}\sec^{2}L)v_{e}$	$F(6,1)=-f_{n}F(6,2)=f_{e}$	
$F(6,4)=2(w_{ie}\cos L+\frac{v_{e}}{R_{n}+h})$	$F(6,5)=2\frac{v_{n}}{R_{m}+h}$	
$F(6,7)=-2w_{ie}v_{e}\sin L$	$F(7,5)=\frac{1}{R_{m}+h}$	
$F(8,4)=\frac{\sec L}{R_{n}+h}$	$F(8,7)=\frac{v_{e}\tan L}{(R_{n}+h)\cos L}$	F(9,6)=1

#### A.2. Second Matrix ${\left(F_{M}\right)}_{6\times 6}$

$$F_{M}=Diag\left[\begin{array}{cccccc} -\frac{1}{T_{rx}} & -\frac{1}{T_{ry}} & -\frac{1}{T_{rz}} & -\frac{1}{T_{ax}} & -\frac{1}{T_{ay}} & -\frac{1}{T_{az}} \end{array}\right]$$

where $T_{i}$ denotes the correlation time of a Gauss–Markov process.

#### A.3.Third Matrix ${\left(F_{G}\right)}_{2\times 2}$

$$F_{G}=\left[\begin{array}{cc} 0 & 1\\ 0 & -\frac{1}{T_{r}} \end{array}\right]$$

where $T_{r}$ is the correlation time of a Gauss-Markov process.

#### A.4. Fourth Matrix $\Gamma_{I}(t)$

$$\Gamma_{I}(t)=\left[\begin{array}{cc} 0_{3\times 3} & 0_{3\times 3}\\ {\left(C_{b}^{n}\right)}_{3\times 3} & 0_{3\times 3}\\ 0_{3\times 3} & {\left(C_{b}^{n}\right)}_{3\times 3} \end{array}\right]$$

where ${\left(C_{b}^{n}\right)}_{3\times 3}$ denotes the rotation matrix from the b-frame to the n-frame.

### B. Measurement Mode

As described in Equation (3) in Section 2, the measurement mode of the tightly-coupled integration system is:

$$Z(t)=\left[\begin{array}{l} Z_{\rho }(t)\\ Z_{\dot{\rho }}(t) \end{array}\right]=\left[\begin{array}{l} H_{\rho }\\ H_{\dot{\rho }} \end{array}\right]X(t)+V(t)$$

where $H_{\rho }={\left[O_{4\times 6}\vdots H_{\rho 1}\vdots O_{4\times 6}\vdots H_{\rho 2}\right]}_{4\times 17}$, $H_{\rho 1}=\left[\begin{array}{ccc} a_{11} & a_{12} & a_{13}\\ a_{21} & a_{22} & a_{23}\\ a_{31} & a_{32} & a_{33}\\ a_{41} & a_{42} & a_{43} \end{array}\right]$, $H_{\rho 2}=\left[\begin{array}{cc} 1 & 0\\ 1 & 0\\ 1 & 0\\ 1 & 0 \end{array}\right]$ $H_{\dot{\rho }}={\left[O_{4\times 3}\vdots H_{\dot{\rho }1}\vdots O_{4\times 9}\vdots H_{\dot{\rho }2}\right]}_{4\times 17}$, $H_{\dot{\rho }1}=\left[\begin{array}{ccc} b_{11} & b_{12} & b_{13}\\ b_{21} & b_{22} & b_{23}\\ b_{31} & b_{32} & b_{33}\\ b_{41} & b_{42} & b_{43} \end{array}\right]$, $H_{\dot{\rho }2}=\left[\begin{array}{cc} 0 & 1\\ 0 & 1\\ 0 & 1\\ 0 & 1 \end{array}\right]$

The factors $a_{ij}$ and $b_{ij}$ re computed as:

$$\begin{array}{c} a_{j1}=(R_{n}+h)[-e_{j1}\sin L\cos\lambda -e_{j2}\sin L\sin\lambda ]\\ \;+[R_{n}(1-e^{2})+h]e_{j3}\cos L\\ a_{j2}=(R_{n}+h)[e_{j2}\cos L\cos\lambda -e_{j1}\cos L\sin\lambda ]\\ a_{j3}=e_{j1}\cos L\cos\lambda +e_{j2}\cos L\sin\lambda +e_{j3}\sin L \end{array}$$

$$\begin{array}{c} b_{j1}=-e_{j1}\sin\lambda +e_{j2}\cos\lambda \\ b_{j2}=-e_{j1}\sin L\cos\lambda -e_{j2}\sin L\sin\lambda +e_{j3}\cos L\\ b_{j3}=e_{j1}\cos L\cos\lambda +e_{j2}\cos L\sin\lambda +e_{j3}\sin L \end{array}$$
